# Severe, uncontrolled epilepsy in pregnancy: A population-based case-control study

**DOI:** 10.3310/nihropenres.13743.1

**Published:** 2024-09-24

**Authors:** Bryn Kemp, Andrew Kelso, David Williams, Marian Knight

**Affiliations:** 1 Maternity Department, Royal Berkshire Hospitals NHS Foundation Trust, Reading, RG1 5AN, UK; 2 NHS Suffolk and North East Essex ICB, Ipswich, IP1 2BX, UK; 3 Elizabeth Garrett Anderson Institute for Women’s Health, University College London Hospitals NHS Foundation Trust, London, NW1 2BU, UK; 4 National Perinatal Epidemiology Unit, Nuffield Department of Population Health, University of Oxford, Old Road Campus, Oxford, OX3 7LF, UK

**Keywords:** Epilepsy, management, outcomes, maternal morbidity, UK Obstetric Surveillance System

## Abstract

**Background:**

Epilepsy affects one percent of the UK population and is the most common serious neurological condition experienced during pregnancy. We compared the characteristics, clinical management, and pregnancy outcomes in women with severe, uncontrolled epilepsy to those of women with well controlled disease.

**Methods:**

We conducted a population-based case-control study in all UK consultant-led maternity units. Cases of severe uncontrolled epilepsy during pregnancy were identified prospectively and reported via the UK Obstetric Surveillance System (UKOSS). Severe epilepsy was defined *a-priori* as ≥1 of the following: admission to hospital during pregnancy to manage seizures; prescribed ≥3 antiepileptic medications; or died from epilepsy. Controls comprised women with epilepsy not meeting the case definition, identified within the same centres as cases. Pre-pregnancy epilepsy control and pregnancy outcomes were compared between groups using multivariable logistic regression.

**Results:**

We identified 94 cases between 1 October 2015 and 31 March 2017 and compared these with 186 controls. Cases were significantly more likely to be admitted to manage seizures in the year preceding pregnancy (42/94 cases vs 10/186 controls, adjusted odds ratio [aOR]=7.38 [95% CI 2.70-20.2]), and to report their most recent seizure within 3 months of pregnancy (51/94 cases vs 18/186 controls, aOR=5.86 [95% CI 2.30-15.0]). Cases were significantly more likely to deliver before 37 weeks (20/94 cases vs 8/186 controls, aOR=7.61 [95% CI 2.87-20.2]).

**Conclusions:**

Women admitted for seizure management in the year before pregnancy are at higher risk of severe epilepsy during pregnancy and of preterm birth. These women should be prioritised for discussion about pregnancy and contraception. When pregnant, they should be reviewed as early as possible by specialists in the management of epilepsy during pregnancy. Delivering messages about the importance of pregnancy planning and contraception to all women with epilepsy should be viewed as the responsibility of all clinicians involved their care.

## Plain Language Summary

Epilepsy is the most common serious neurological condition that women experience during pregnancy. We wanted to understand how women who have severe, uncontrolled epilepsy during pregnancy differ from those with well-controlled epilepsy. We also investigated whether these two groups received different clinical care during pregnancy, and how they and their babies fared during and after birth.

The UK Obstetric Surveillance System (UKOSS) gathers anonymous information from all UK maternity units about pregnant women with certain conditions. We used UKOSS to collect information about women with severe, uncontrolled epilepsy during pregnancy. The authors confirm that this collection of Information was approved by UKOSS and the London Multi-Centre Research Ethics Committee. We defined this group as pregnant women who:

- were prescribed three or more anti-epileptic medications, or
- were admitted to hospital because of seizures, or
- died of epilepsy.

We compared these women with a group of pregnant women with epilepsy who did not meet any of these criteria.

The women with severe, uncontrolled epilepsy were more likely to have been admitted to hospital because of seizures in the year before they got pregnant. They were also more likely to have had their most recent seizure 3 months or less before they got pregnant. Women with severe, uncontrolled epilepsy were more likely than women with well-controlled epilepsy to give birth early, before 37 weeks; 20% of these early births were spontaneous, while 80% were recommended by clinicians.

Women with epilepsy who have been to hospital with a seizure in the year before getting pregnant are at greater risk of severe epilepsy during pregnancy and of giving birth early. These women should be seen by epilepsy specialists as a priority, as early as possible during pregnancy. All healthcare professionals caring for women of childbearing age with epilepsy should be aware that it is important to discuss contraception and pregnancy planning, particularly with non-pregnant women who come to hospital with seizures.

## Introduction

Epilepsy affects around one percent of the UK population and is the most common serious chronic neurological condition in pregnancy ^1, 2^. Although birth outcomes for most affected women are usually good, a significant minority of pregnant women are at risk of epilepsy-related morbidity and mortality ^3– 5^. In the UK between 2019 and 2021, the mortality rate for women who died from causes related to epilepsy during or up to a year after the end of pregnancy was 0.76 per 100,000 maternities (95% confidence interval [CI] 0.44–1.22) ^2^. Assuming only approximately 1% of the population have epilepsy ^1^, this equates to 1 maternal death related to epilepsy for every 1300 pregnant women with epilepsy. The lack of improvement in this rate over time is a concern; In 2013–15 the mortality rate was 0.52 per 100,000 maternities (95% CI 0.28–0.8) ^6^ and in 2016–2018, 0.91 per 100,000 maternities (95% CI 0.57–1.38) ^7^. The need to improve birth outcomes for women with epilepsy is therefore a national priority ^6, 8, 9^.

While the precise mechanisms underlying the poor pregnancy outcomes observed in some women with epilepsy are unclear, they are likely to be multifactorial. In the most extreme cases, ending in maternal mortality, review as part of confidential enquiries has consistently highlighted ‘Sudden Unexplained Death in EPilepsy’ (SUDEP) as a central component in the final common pathway leading to death. As a diagnosis of exclusion, characterized by the persistence of uncontrolled, convulsive seizures, SUDEP can only be attributed where no other cause of death is identified at autopsy ^10– 12^. It was implicated in the cases of 14 of the 17 women who died of causes related to epilepsy during pregnancy or up to a year after birth in the UK between 2019 and 2021 (mortality rate 0.63 per 100,000 maternities, 95% CI 0.34–1.05) ^2^.

Women who are seizure-free for at least a year before conceiving are likely to remain so during pregnancy, and the risk of seizures during pregnancy decreases with increasing duration of the preconception seizure-free period ^5, 12– 14^. Conversely, experiencing uncontrolled convulsive seizures immediately prior to pregnancy was identified in all but one maternal death attributed to epilepsy between 2013 and 2015 when reviewed as a case series by the UK’s Confidential Enquiry into maternal mortality ^6^. Therefore, achieving a steady-state that is free from convulsive seizures in the time leading up to pregnancy seems a logical treatment goal. The process towards improving outcomes for this group must begin with a clearer understanding of the characteristics of pregnancies in women with severe, uncontrolled epilepsy. Such an understanding should be developed by comparison to those with well controlled disease, rather than healthy controls, so that universal changes in the management of all pregnant women with epilepsy can be avoided. To this end, policy makers and healthcare providers have called on the research community to prioritise high-quality, prospective research as a basis for improving the outcomes of pregnant women with epilepsy ^10, 13, 15– 17^.

The primary aims of this study were to describe the characteristics and pregnancy outcomes of women with severe, uncontrolled epilepsy using the United Kingdom Obstetric Surveillance System (UKOSS) and to compare outcomes between this group of women and a control group of women with epilepsy.

## Methods

### Patient and public involvement

Patients were not directly involved in the design of this study. Two members of the public were indirectly involved in the design of the study as representatives on the UKOSS Steering Committee, which reviews, comments on, and approves the choice of conditions to be investigated and the design and data collection forms for all studies to be run through UKOSS. Patients and the public were not involved in dissemination plans for the study.

### Study population and design

A nationwide unmatched population-based case-control study was performed identifying women with severe, uncontrolled epilepsy delivering in all 199 consultant-led maternity units in the UK between 1 October 2015 and 31 March 2017, inclusive. With no established definition for ‘severe and uncontrolled epilepsy’, a pragmatic case definition was used, including 1) any woman with epilepsy who died during pregnancy or up to day 42 after delivery, where the cause of death was directly attributed to the consequences of epilepsy, including SUDEP; 2) any woman admitted to hospital for management of generalised tonic-clonic seizures during pregnancy or the post-partum period; or 3) any woman being treated with 3 or more antiepileptic drugs simultaneously during pregnancy.

An unmatched control group comprising women with epilepsy not meeting the case definition was obtained from all UK maternity units. Control data were requested from the first one, two or three women with epilepsy (stratified by size of unit) delivering in the month of September 2016 who did not meet the case definition.

Based on the final data, the study had 80 percent power (α=0.05) to detect odds ratios (ORs) of 2.14 and 5.90 for the most prevalent (convulsive pre-pregnancy seizure type) and least prevalent (co-existing mental health diagnosis) risk factors amongst the control group, respectively.

### UKOSS

UKOSS is a national maternity research platform designed to examine specific rare but clinically important conditions in pregnancy in the UK. The active negative surveillance methods have been described in detail in previous publications ^18^. Briefly, nominated reporters at each centre notified the UKOSS coordinating centre on a monthly basis about any women meeting the inclusion criteria for surveillance conditions admitted to their unit. Notifying clinicians were then sent anonymised study-specific data collection forms and asked to provide data on cases. The data collection form for this study can be accessed here: https://www.npeu.ox.ac.uk/assets/downloads/ukoss/forms/UKOSS-Epilepsy-V1.pdf. Once returned to the UKOSS coordinating centre, data were double-entered into a study-specific database, validated, and checked in preparation for analysis. Queries were directed back to reporting centres for clarification and a system involving up to five reminders was used to maximise the completeness and accuracy of the study dataset. Data for controls were requested as previously described.

### Statistical methods

The associations between pre-specified characteristics relating to the sociodemographic, pre-pregnancy epilepsy, pregnancy (including epilepsy during pregnancy), and labour and delivery characteristics were examined using logistic regression. The potential for multi-collinearity between exposure variables was assessed using Pearson’s correlation coefficient. The outcomes reported in the study are consistent with the ‘E-CORE’ core outcome framework recommendations for studies on epilepsy in pregnancy ^15^.

A forward stepwise regression method was used to explore the relationship between multiple exposure variables and case status as the outcome of interest. Those characteristics associated with poor outcomes in pregnancy identified from existing literature as well those with a *p*-value <0.1 in the univariate analyses were considered as candidate variables. Characteristics were ordered according to their temporal relationship with delivery with those at the proximal extreme, and so the greatest time interval between their occurrence and delivery, were entered first. Results are summarised using adjusted odds ratios (aORs) and associated 95% confidence intervals (95% CIs). Epilepsy characteristics observed during the index pregnancy or labour and delivery were excluded from the multivariable model on the grounds that any statistical associations may be because of the case definition itself, rather than a reflection of more severe disease.

Pregnancy outcomes were explored using separate models. For analysis purposes, maternal morbidity was defined by episodes of end-organ failure, acute kidney injury, the need for admission to intensive care >24 hours, or delivery at <37 weeks’ gestation for maternal indications. Analyses were adjusted for sociodemographic status (maternal age and employment status), coexisting hypertensive disease (pre-existing and pregnancy induced), diabetes (pre-existing and gestational), and other ‘significant pregnancy problems’ as potential confounders. The association between case status and newborn morbidity (NICU admission, APGAR <5 at 5 minutes of age) was examined with adjustment for mode of birth, gestational age at birth, birthweight <10 ^th^ centile for gestational age, and maternal sociodemographic status (maternal age and employment status).

It has been shown previously that the distribution of missing data within UKOSS datasets is not random ^18^. Consequently, multiple imputation was avoided, and missing data categories were included in the initial univariate comparisons. The potential for bias relating to such data to adversely affect estimates of association between exposures and case status was explored by recoding missing data into the extreme categories of respective exposure variables and reconstructing the adjusted regression models. All statistical analyses were performed using STATA v15.0 ^19^.

### Sensitivity analyses

Because there is no established definition for ‘severe and uncontrolled epilepsy’, we used a pragmatic case definition consisting of three elements. To investigate whether there was any evidence of differential outcomes according to whether women were managed with three or more anti-epileptic drugs or whether they were admitted for control of generalised tonic-clonic seizures, we conducted sensitivity analyses examining outcomes in the group of cases restricted only to those who were managed with three or more anti-epileptic drugs and to those who were admitted for control of generalised tonic-clonic seizures.

### Ethical approval

The UK Obstetric Surveillance System general methodology was approved by the London Multi-Centre Research Ethics Committee (04/MRE02/45; 24 September 2004) and ethics approval for the current study as a substantial amendment was granted by the North London REC1 (study reference 10/H717/20; 19 August 2015). The UKOSS methodology involves collection of information only, for the purpose of studying incidence and identifying means to improve patient care, which is not individually identifiable and does not lead to any change in management for the individual patient. In these circumstances, individual patient consent is not required. The ethical committee approved this position. Therefore, consent was not required for the collection of anonymous routine data.

### Role of the funding source

The funders of the study had no role in study design, data collection, data analysis, data interpretation, or writing of the manuscript. The corresponding author had full access to all the data in the study and had final responsibility for the decision to submit for publication.

## Results

**Figure 1.**
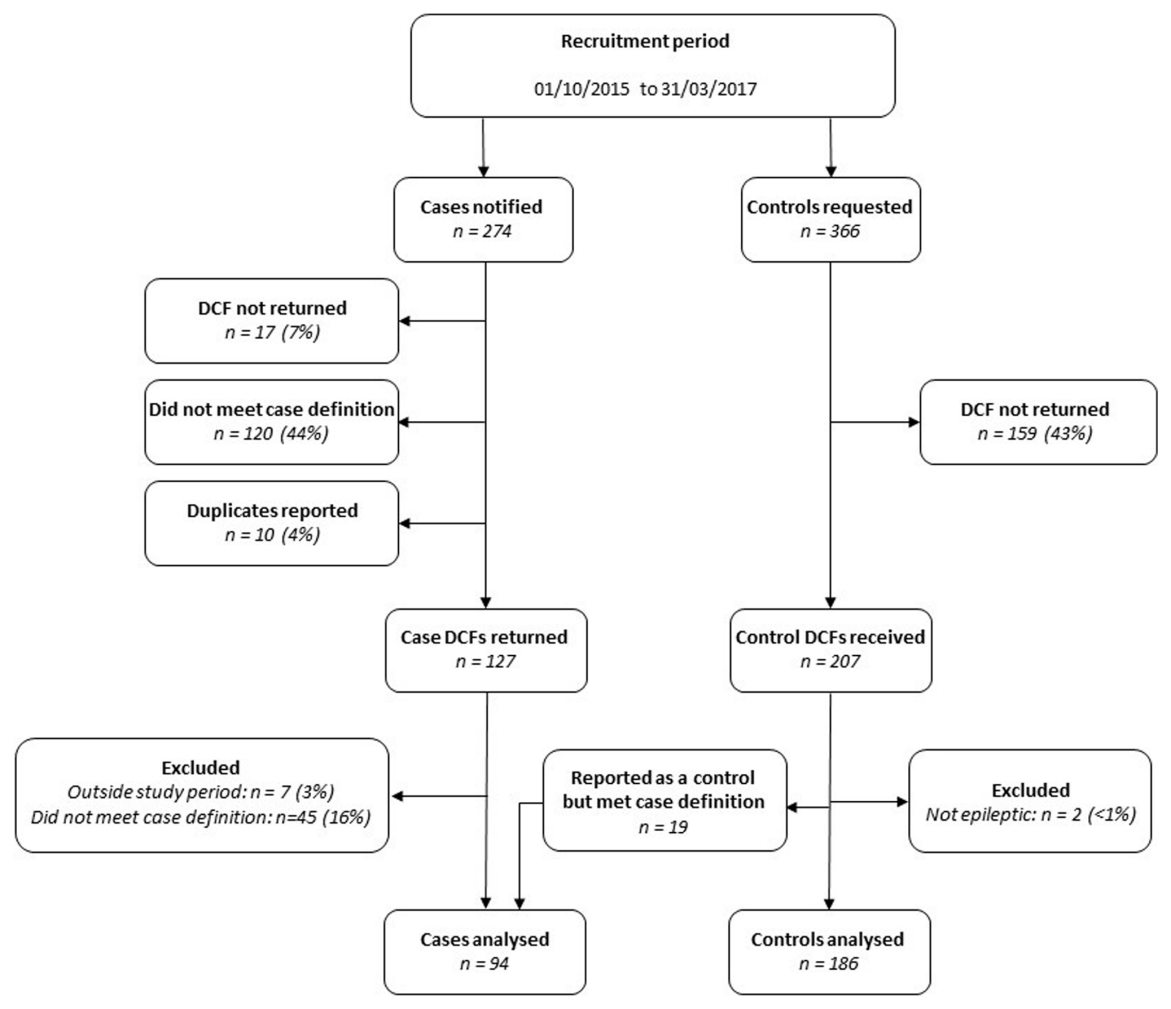
Study participants. DCF, data collection form.

All consultant-led maternity units in the UK participated with UKOSS notification during the study period, identifying 94 cases and 186 controls in total. Although 274 notifications were made through the UKOSS case notification system, 165 (60%) did not meet the case definition; the majority of these women had epilepsy but were not being treated with three or more anti-epileptic drugs and had been admitted to hospital for reasons other than control of their epilepsy. Amongst the group of controls, 19 (9%) were subsequently found to meet the case definition, so were transferred into the case group for analysis ( Figure 1).

### Characteristics of cases and controls

The final group of 94 cases included 3 (3.2%) women who died from epilepsy-related causes, 56 (60.0%) women who were admitted to hospital to manage generalised tonic-clonic seizures, 20 (21.3%) women who were prescribed ≥3 antiepileptic medications and 15 (16.0%) women who were admitted and also used ≥3 medications. Of the three women in the case group who died, 2 deaths were attributed to SUDEP and 1 to drowning on a background of SUDEP. There were no maternal deaths in the control group. Ninety-two of 94 cases (97.9%) and 184/186 (98.9%) controls had been diagnosed with epilepsy prior to the index pregnancy, with a median time since diagnosis of 12.3 years (IQR 8.4–19.2) and 15.2 years (IQR 9.2–21.2) for cases and controls, respectively.

Table 1, Table 2, Table 3 and Table 4 show the sociodemographic characteristics, pre-pregnancy epilepsy characteristics, index pregnancy characteristics (including epilepsy during pregnancy), and the labour and delivery characteristics of cases and controls, respectively. Two characteristics were found to have a statistically significant association with case status after adjustment: hospital admission to manage seizures in the year before pregnancy (aOR = 7.38 [95% CI 2.70–20.2]), and experiencing the most recent seizure within 3 months of the index pregnancy (aOR = 5.86 [95% CI 2.30–15.0]) ( Table 5). 

**Table 1.** Sociodemographic characteristics of cases and controls.

Characteristic	Cases (n=94)		Controls (n=186)		Unadjusted OR	95% Confidence Interval
	*n*	*%*	*n*	*%*		
**Age (years)**						
<25	22	23.4	20	10.8	2.16	1.08–4.30
26–35	53	56.4	104	55.9	1	
>35	18	19.1	61	33.2	0.27	0.12–0.60
Missing	1	1.1	1	0.5		
**Marital status**						
Single	29	30.9	41	22.3	1.56	0.89–2.72
Married/cohabiting	65	69.1	143	77.7	1	
Missing			2	0.01		
**Ethnic group**						
Black or other minority ethnic group	12	12.8	33	17.7	0.67	0.33–1.37
White	82	87.2	151	81.2	1	
Missing			2	1.1		
**Employed**						
Yes	33	35.1	121	64.7	1	
No	57	60.6	62	33.2	3.37	1.99–5.71
Missing	4	4.3	3	1.6		
**Body Mass Index (kg/m ^2^)**						
<25	42	44.7	77	41.2	1	
25–29.9	19	20.2	59	31.6	0.58	0.31–1.12
30–34.9	23	24.5	40	21.4	1.05	0.56–1.99
>35	5	5.3	7	3.7	1.31	0.39–4.38
Missing	5	5.3	3	1.6		
**Smoking status**						
Non-smoker	76	80.9	153	81.8	1	
Smoked during pregnancy	16	17.0	31	16.6	1.04	0.54–2.02
Missing	2	2.1	2	1.1		

OR, odds ratio

**Table 2.** Pre-pregnancy epilepsy characteristics of cases and controls.

Characteristic	Cases (n=94)		Controls (n=186)		Unadjusted OR	95% Confidence Interval
	*n*	*%*	*n*	*%*		
**Epilepsy before current pregnancy**						
Yes	92	97.9	184	98.9	1	
No	2	2.1	2	1.1	2.01	0.28–14.5
**Most recent seizure before pregnancy**						
Within 3 months	51	54.3	18	9.7	16.72	8.17–34.2
3–6 months	8	8.5	12	6.5	3.93	1.43–10.8
6–12 months	3	3.2	13	7	1.36	0.36–5.21
>12 months	20	21.3	118	63.4	1	
Not epileptic	2	2.1	2	1.1		
Missing	10	10.6	23	12.4		
**Hospital admission to manage seizures during the 1 year pre-pregnancy**						
Yes	42	44.7	10	5.4	7.98	3.52–18.1
No	50	53.2	174	93.5	1	
Not epileptic	2	2.1	2	1.1		
**Predominant seizure type during the 1 year pre-pregnancy**						
Convulsive	73	77.7	111	59.7	1.68	0.92–3.04
Other	20	21.3	51	27.4	1	
Missing	1	1.1	24	12.9		
**Epilepsy specialist review - preconception**						
Yes	59	62.8	86	46.2	2.06	1.23–3.44
No	35	37.2	100	53.8	1	
**Known to Epilepsy services at booking**						
Yes	94	100	146	78.5	0.63	0.09–4.58
No	0		35	21.5	1	
Missing			5	2.7		

OR, odds ratio

**Table 3.** Pregnancy characteristics (including epilepsy in pregnancy) of cases and controls.

Characteristic	Cases (n=94)		Controls (n=186)		Unadjusted OR	95% Confidence Interval
	*n*	*%*	*n*	*%*		
**Planned pregnancy**						
Yes	53	56.4	136	73.1	1	
No	41	43.6	50	26.9	2.10	1.25–3.54
**Parity**						
Nulliparous	47	50	86	46.2	1.16	0.71–1.91
Multiparous	47	50	100	53.8	1	
**Late booking (>12/40)**						
Yes	15	16.0	45	24.2	0.60	0.31–1.15
No	76	80.9	136	73.1	1	
Missing	3	3.2	5	2.7		
**Hypertensive disorders of pregnancy**						
Yes	2	2.1	10	5.4	0.38	0.08–1.79
No	92	97.9	176	94.6	1	
**Diabetes (including gestational)**						
Yes	5	5.3	5	2.7	2.04	0.58–7.25
No	89	94.7	181	97.3	1	
**Mental health disorders**						
Yes	5	5.3	4	2.2	2.57	0.67–9.81
No	89	94.7	182	97.8	1	
**Seizures during current pregnancy**						
Yes	87	92.6	35	18.8	54.0	23.0–126.69
No	7	7.4	151	81.2	1	
**Out of hospital seizures during current pregnancy**						
Yes	84	89.4	32	17.2	40.4	18.9 – 86.3
No	10	10.6	154	82.8	1	1
**Predominant seizure type during current pregnancy**						
Status epilepticus	8	8.5	0	0		
Convulsive	56	59.6	12	6.5	4.46	1.90–10.5
Other	23	24.5	22	11.8	1	
Seizure free	7	7.4	151	81.2		
Missing			1	0.5		
**Epilepsy specialist review during current pregnancy**						
Yes	74	78.7	85	45.7	4.35	2.45–7.70
No	20	21.3	101	54.3	1	
**Epilepsy nurse specialist involvement during current pregnancy**						
Yes	63	67.0	87	46.8	2.34	1.39–3.92
No	31	33.0	99	53.2	1	

OR, odds ratio

**Table 4.** Labour and delivery characteristics of cases and controls.

	Cases (n=89) ^1^		Controls (n=183)		Unadjusted OR	95% Confidence Interval
	*n*	*%*	*n*	*%*		
**Seizures during labour and delivery**						
Yes	10	11.2	2	1.1	11.0	2.35–51.1
No	79	88.8	181	98.9	1	
**Type of seizure during labour and delivery (predominant) ^2^**						
Status epilepticus	2	2.3	0	0		
Convulsive	4	4.5	2	1.1		
Other	4	4.5	0	0		
Seizure free	79	88.7	181	98.9		
**Epilepsy specialist review - during labour and delivery**						
Yes	22	24.7	18	9.8	2.85	1.44–5.64
No	67	75.2	165	90.2	1	
**Analgesia for labour delivery**						
None	19	20.2	16	8.6	3.17	1.44–6.95
Regional / PCA	45	47.9	90	48.4	1.33	0.77–2.31
Other	30	31.9	80	43.0	1	
**General anaesthesia for delivery**						
Yes	9	11.2	13	7.1	1.41	0.58–3.43
No	80	89.9	170	92.9	1	
**Obstetric haemorrhage**						
Yes	12	13.5	10	6.5	2.12	0.91–4.93
No	77	86.5	173	94.5	1	
**Delivery induced**						
Yes	43	48.3	70	38.3	1.37	0.83–2.26
No	46	51.7	113	61.8	1	
**Mode of birth**						
Spontaneous vaginal birth	43	48.3	91	49.7	1	
Operative vaginal birth	12	13.5	29	15.9	0.88	0.41–1.88
Emergency Caesarean Section ^3^	8	9.0	25	13.7	0.68	0.29–1.62
Elective Caesarean Section ^4^	26	29.2	37	20.3	1.49	0.80–2.76
Missing			1	0.5		
**Preterm birth <37 weeks’**						
Yes	20	22.5	8	4.4	6.21	2.62–14.8
No	68	76.4	173	95.1	1	
GA missing	1	1.1	2	1.1		
**Birthweight <10 ^th^ centile ^5^**						
Yes	7	7.9	12	6.6	1.21	0.46–3.19
No	82	92.1	171	93.4	1	

^1^Pregnancies ending in miscarriage or termination excluded from this analysis ( *n*=8). ^2^Confidence intervals of OR wide and unstable, therefore omitted. ^3^NICE categories 1 & 2 ^19^. ^4^NICE categories 3 & 4. ^5^Newborn sex-specific centiles calculated using INTERGROWTH-21 ^st^ standards ^14^ OR, odds ratio; PCA, patient-controlled analgesia; GA, gestational age

**Table 5.** Factors associated with severe uncontrolled epilepsy.

Characteristic	Adjusted OR	95% CI
**Age (years)**		
<25	2.35	0.87–6.43
26 – 35	1	
>35	0.70	0.31–1.59
**Employed**		
Yes	1	
No	1.75	0.78–3.91
**Planned pregnancy**		
Yes	1	
No	0.89	0.37–2.10
**Most recent seizure before pregnancy**		
Within 3 months	5.86	2.30–15.0
3–6 months	0.94	0.25–3.55
6–12 months	0.54	0.10–2.91
>12 months	1	
**Admitted to manage seizures during the ** **year prior to pregnancy**		
Yes	7.38	2.70–20.2
No	1	
**Predominant seizure type during the year ** **prior to pregnancy**		
Convulsive	1.85	0.82–4.15
Other	1	
**Epilepsy specialist review – preconception**		
Yes	0.90	0.44–1.82
No	1	

OR, odds ratio; CI, confidence interval

### Pharmacological management

Amongst cases, 6/94 (6.4%) were prescribed no antiepileptic medication during the index pregnancy, with 2 of these 6 (33.3%) reporting convulsive seizures during the year preceding the index pregnancy. In comparison, 54/186 (29.0%) controls were not prescribed antiepileptic medication, of whom 23/54 (42.6%) reported a predominantly convulsive pre-pregnancy seizure type. Levetiracetam was the most widely prescribed agent amongst cases (50/94 [53.2%] *versus* 46/186 [24.7%] controls, *p*<0.001), although lamotrigine was more commonly prescribed overall (48/94 [51.1%] cases and 73/186 [39.3%] controls, *p*=0.0598). Sodium valproate was prescribed to 10/94 (10.6%) cases and 12/186 (6.5%) controls, although the difference in proportions was not statistically significant ( *p*=0.2291). Phenytoin, perampanel, lacosamide, gabapentin, and ethosuximide were used in cases but not in controls. After giving birth, 37/94 (40.7%) cases and 55/186 (29.6%) controls were counselled about contraception ( *p*=0.0628).

### Pregnancy outcomes

Two hundred and seventy infants were liveborn (96.3%), 2 infants of cases were stillborn and 8 (3.7%) pregnancies miscarried or were terminated (5 cases and 3 controls). Amongst pregnancies reaching viability (n=272), the odds of being born preterm (<37 weeks) were significantly raised for cases, with 20/89 (22.5%) and 8/183 (4.4%) preterm deliveries amongst cases and controls, respectively (aOR 7.61, 95% CI 2.87–20.2). There were no statistically significant differences in the odds of induction of labour, mode of birth, or infants born <10 ^th^ centile for gestational age ^20^.

Suspected fetal abnormalities were identified during routine ultrasound screening in 1/94 (1.1%) cases and 7/186 (3.8%) controls, with 1 case and 5 controls having anomalies confirmed at delivery, respectively. Abnormalities included chromosomal, cardiac, craniofacial and musculoskeletal defects. No information was available on causality relating to anti-epileptic drugs.

Although there were higher odds of morbidity for cases and their newborns ( Table 6), these associations did not reach statistical significance in adjusted analyses. There were no material differences in the results if the cases were limited either to women managed with three or more anti-epileptic drugs or to women who were admitted for control of generalised tonic-clonic seizures (data not shown). Preterm birth was spontaneous in 4 of the 20 cases who delivered at <37 weeks (20%), with the remaining 16/20 (80%) preterm births in cases occurring following decisions by attending clinicians to recommend early delivery [9/16 (56.2%) for presumed maternal compromise, in the majority of cases relating to their epilepsy; 3/16 (18.8%) for suspected fetal growth restriction, and no reason provided in 4/16 (25%) of elective preterm births].

**Table 6.** Maternal and newborn clinical outcomes.

Outcome	Cases (n=89) ^1^		Controls (n=183)		Unadjusted OR (95% CI)	Adjusted OR (95% CI)
	*n*	*%*	*n*	*%*		
**Preterm birth <37 weeks ^2^**						
Yes	20	22.5	8	4.4	6.21 (2.62–14.8)	7.61 (2.87–20.2)
No	69	77.5	175	95.6	1	1
**Maternal morbidity ^3^**						
Yes	7	8.9	5	2.7	2.91 (0.90–9.44)	3.17 (0.86–11.7)
No	82	92.1	178	97.3	1	1
**Newborn morbidity ^2^**						
Yes	20	22.5	13	7.1	3.73 (1.79–7.76)	2.56 (0.95–6.92)
No	69	77.5	170	92.9	1	1

^1^Pregnancies ending in miscarriage or termination excluded (n=8). ^2^Adjusted for maternal age, employment status, mode of birth, gestational age at birth, confirmed congenital abnormality and birthweight<10 ^th^ centile for gestational age. ^3^Adjusted for maternal age, employment status, hypertensive disease, diabetes and other 'significant pregnancy problems'. OR, odds ratio; CI, confidence interval

## Discussion

### Main findings

Two characteristics were independently associated with ‘severe and uncontrolled epilepsy’: the need for admission to manage seizures in the year before pregnancy and having had the most recent seizure in the 3 months immediately prior to the index pregnancy. The likelihood of delivery at <37 weeks was significantly higher in cases than in controls. Although there were no statistically significant differences with respect to maternal or newborn outcomes between the groups, the odds of most adverse outcomes were higher in cases than controls. SUDEP was implicated in the final cause of death for all three women who died.

Almost all women had a diagnosis of epilepsy at booking and most were already known to epilepsy specialist services. Despite such an encouraging finding, over one third of cases and more than half of controls had no documented review with an epilepsy specialist prior to the index pregnancy, which was unplanned in 44% of cases and 27% of controls. Management plans showed no evidence that post-delivery counselling about the importance of effective contraception had taken place in over half of cases and more than two thirds of controls. The introduction of the Valproate Pregnancy Prevention Programme ^21^ and Maternal Medicine Networks in England ^22^ since this study was conducted may have altered this but it is nevertheless a concern.

### Strengths and limitations

The UKOSS platform underpins the strengths associated with this study. This nationwide system provides active surveillance embedded within each of the obstetric units in the UK and so is well placed to collect prospective data on rare outcomes at a population level; thus minimising bias that can be associated with studies based in a single centre.

The study’s clinical case definition was developed by a team of obstetric, neurology and public health clinicians with the intention that high-risk pregnancies could be identified from within routine clinical settings and without the need for complex diagnostic testing. The definition was difficult to use in practice for prospective case detection, which resulted in nine percent of those women reported as controls being found, subsequently, to satisfy the case definition. This difficulty appeared to be linked predominantly to a problem attributing the primary reason for antenatal admissions to convulsive seizure activity, which raises the possibility of incomplete case identification amongst the group analysed, and was also the reason why 44% of notifications were found to not meet the case definition. The small study size, which was driven by disease incidence, makes it possible that clinically important differences between cases and controls were not detected as statistically significant.

## Interpretation

Overall, the results from this study point towards the preconception period as a critical window in which to optimise the management of epilepsy. With an important proportion of those with a diagnosis of epilepsy being women of child-bearing age, it is neither feasible nor realistic to expect a single group of healthcare professionals to be responsible for communicating the importance of optimizing seizure control as part of wider pregnancy planning ^23^. However, these messages can be integrated in to the care pathways for women with epilepsy within primary care, emergency departments and early pregnancy units as well as maternity care and neurology services.

Despite existing literature suggesting that there are increased rates of pre-eclampsia, gestational diabetes, caesarean section, and obstetric haemorrhage at delivery, when women with epilepsy were compared to healthy control groups, the same findings were not replicated when comparing cases and controls in this study ^16, 24, 25^. Rates of pre-eclampsia and gestational diabetes were similar to those observed in the general population ^26, 27^. This study did, however, observe similar rates of preterm birth and small for gestational age newborns to those reported elsewhere ^13^. As may be expected, cases had more seizures, which were most likely to be convulsive in nature, during pregnancy, labour, and delivery and so it is reassuring that these women were also more likely to have been reviewed by epilepsy specialists.

Despite a higher risk of preterm delivery at <37 weeks’ gestation, there were no statistically significant differences in maternal or newborn morbidity outcomes for cases, which may reflect limited power to detect such associations due to the small study size. Whatever the reasons for deciding to deliver individual women preterm, such decisions are associated with a predictable increase in risks to the newborn because of gestational age-related complications. For this reason, preterm birth at <37 weeks should be viewed as an important consequence of severe disease that, itself, is amenable to reduction by improvements in epilepsy control before pregnancy.

The results of this study highlight the importance of clinicians being vigilant of the potential for the risks of epilepsy-related morbidity to change as pregnancy progresses. There were several examples of women in early pregnancy reporting characteristics that would be considered as being protective against adverse outcomes, such as being seizure free for more than a year, who then went on to experience a recurrence of seizures leading to their inclusion during the study as a case. Such instances illustrate the importance of review of all women by epilepsy specialists to highlight disease-specific factors that may influence risk at the individual pregnancy level.

## Conclusion

Using a pragmatic case definition of severe epilepsy in pregnancy, this study highlights the preconception period as a critical time during which improvements in seizure control may have the potential to improve outcomes for pregnant women with epilepsy. Women admitted for seizure management in the year prior to pregnancy are at significantly higher risk of severe epilepsy during pregnancy and of preterm birth. Non-pregnant women of reproductive age admitted for seizure management should be prioritised for discussion of pregnancy planning and contraception. This group should be reviewed as a priority by epilepsy specialists as early as possible during pregnancy. Delivering messages about the importance of pregnancy planning and contraception to all women with epilepsy is important and should be viewed as the responsibility of all clinicians involved their care.

## Data Availability

Data cannot be shared openly because of confidentiality issues and the potential identifiability of sensitive data. Requests to access the data can be made by contacting the National Perinatal Epidemiology Unit data access committee via general@npeu.ox.ac.uk. The Research Ethics Committee approved the study on the basis that access will only be allowed after review of the request by the UK Obstetric Surveillance System Steering Committee. The estimated response time for requests is 4 weeks. Data sharing outside the UK or European Union may require consultation with the UK Health Research Authority. For more information, please refer to the National Perinatal Epidemiology Unit Data Sharing Policy available at https://www.npeu.ox.ac.uk/assets/downloads/npeu/policies/Data_Sharing_Policy.pdf. For the purpose of open access, the authors have applied a Creative Commons Attribution (CC BY) licence to any Author Accepted Manuscript version arising.

## Software availability

All statistical analyses were performed using STATA v15.0, a commercial software. All analyses in this study can be replicated using freely available statistical software such as R.

## References

[1] Wigglesworth S Neligan A Dickson JM : The incidence and prevalence of epilepsy in the United Kingdom 2013-2018: a retrospective cohort study of UK primary care data. *Seizure.* 2023;105:37–42. 10.1016/j.seizure.2023.01.003 36702018

[2] Knight M Bunch K Felker A, (Eds.) : Saving lives, improving mothers’ care core report - Lessons learned to inform maternity care from the UK and Ireland Confidential Enquiries into Maternal Deaths and Morbidity 2019-21.Oxford: National Perinatal Epidemiology Unit, University of Oxford,2023; [accessed 9 May 2024]. Reference Source

[3] Adab N Kini U Vinten J : The longer term outcome of children born to mothers with epilepsy. *J Neurol Neurosurg Psychiatry.* 2004;75(11):1575–83. 10.1136/jnnp.2003.029132 15491979 PMC1738809

[4] Christensen J Vestergaard C Hammer Bech B : Maternal death in women with epilepsy: smaller scope studies. *Neurology.* 2018;91(18):e1716–e1720. 10.1212/WNL.0000000000006426 30258019 PMC6207413

[5] Sveberg L Svalheim S Tauboll E : The impact of seizures on pregnancy and delivery. *Seizure.* 2015;28:35–8. 10.1016/j.seizure.2015.02.020 25746572

[6] Knight M Nair M Tuffnell D, (Eds) : Saving lives, improving mothers’ care - Lessons learned to inform maternity care from the UK and Ireland Confidential Enquiries Into Maternal Deaths and Morbidity 2013-15.Oxford: National Perinatal Epidemiology Unit, University of Oxford,2017; [accessed 9 May 2024]. Reference Source

[7] Knight M Bunch K Tuffnell D, (Eds.) : Saving lives, improving mothers’ care - Lessons learned to inform maternity care from the UK and Ireland Confidential Enquiries into Maternal Deaths and Morbidity 2016-18.Oxford: National Perinatal Epidemiology Unit, University of Oxford,2020; [accessed 9 May 2024]. Reference Source

[8] Knight M Kenyon S Brocklehurst P : Saving lives, improving mother’s care.Oxford: National Perinatal Epidemiology Unit, University of Oxford,2014. Reference Source

[9] Knight M Nair M Tuffnell D : Saving lives, improving mothers’ care-Surveillance of maternal deaths in the UK 2012–14 and lessons learned to inform maternity care from the UK and Ireland Confidential Enquiries into Maternal Deaths and Morbidity 2009–14.National Perinatal Epidemiology Unit, University of Oxford,2017. Reference Source

[10] Edey S Moran N Nashef L : SUDEP and epilepsy-related mortality in pregnancy. *Epilepsia.* 2014;55(7):e72–4. 10.1111/epi.12621 24754364

[11] Langan Y Nashef L Sander JW : Case-control study of SUDEP. *Neurology.* 2005;64(7):1131–3. 10.1212/01.WNL.0000156352.61328.CB 15824334

[12] Royal College of Obstetricians and Gynaecologists: Epilepsy in pregnancy: green-top guideline no. 68.2016; [accessed 10 May 2024]. Reference Source

[13] Harden CL Hopp J Ting TY : Management issues for women with epilepsy-focus on pregnancy (an evidence-based review): I. Obstetrical complications and change in seizure frequency: report of the Quality Standards Subcommittee and Therapeutics and Technology Assessment Subcommittee of the American Academy of Neurology and the American Epilepsy Society. *Epilepsia.* 2009;50(5):1229–36. 10.1111/j.1528-1167.2009.02128.x 19496807

[14] Vajda FJE Hitchcock A Graham J : Seizure control in antiepileptic drug-treated pregnancy. *Epilepsia.* 2008;49(1):172–6. 10.1111/j.1528-1167.2007.01412.x 18031551

[15] Al Wattar BH Tamilselvan K Khan R : Development of a core outcome set for epilepsy in pregnancy (E-CORE): a national multi-stakeholder modified Delphi consensus study. *BJOG.* 2017;124(4):661–667. 10.1111/1471-0528.14430 27860117

[16] Battino D Tomson T Bonizzoni E : Seizure control and treatment changes in pregnancy: observations from the EURAP epilepsy pregnancy registry. *Epilepsia.* 2013;54(9):1621–7. 10.1111/epi.12302 23848605

[17] National Institute for Health Care Excellence: Epilepsies: diagnosis and management.2012; [accessed 20/11/2018]. Reference Source

[18] Lindquist A Knight M Kurinczuk JJ : Variation in severe maternal morbidity according to socioeconomic position: a UK national case-control study. *BMJ Open.* 2013;3(6): e002742. 10.1136/bmjopen-2013-002742 23794588 PMC3686175

[19] StataCorp: Stata Statistical Software: Release 15. College Station, TX: StataCorp LLC,2017. Reference Source

[20] Villar J Ismail LC Victora CG : International standards for newborn weight, length, and head circumference by gestational age and sex: the Newborn Cross-Sectional Study of the INTERGROWTH-21 ^st^ Project. *Lancet.* 2014;384(9946):857–68. 10.1016/S0140-6736(14)60932-6 25209487

[21] Medicines and Healthcare Products Regulatory Agency: Drug Safety Update. Valproate Pregnancy Prevention Programme: actions required now from GPs, specialists, and dispensers.2018;12(2). [accessed 11 June 2024]. Reference Source

[22] NHS England: Maternal medicine network service specification. Version 1,2021; [accessed 11 June 2024]. Reference Source

[23] National Institute for Health and Care Excellence: Epilepsies in children, young people and adults [NG217].2022; [accessed 13 May 2024]. Reference Source 35700280

[24] Razaz N Tomson T Wikström AK : Association between pregnancy and perinatal outcomes among women with epilepsy. *JAMA Neurol.* 2017;74(8):983–991. 10.1001/jamaneurol.2017.1310 28672292 PMC5710333

[25] Viale L Allotey J Cheong-See F : Epilepsy in pregnancy and reproductive outcomes: a systematic review and meta-analysis. *Lancet.* 2015;386(10006):1845–52. 10.1016/S0140-6736(15)00045-8 26318519

[26] NHS England: Recommendations for digital blood pressure monitoring in maternity services.2023; [accessed 31 July 2024]. Reference Source

[27] National Institute for Health and Care Excellence: Diabetes in pregnancy: management from preconception to the postnatal period.2015; (updated December 2020), [accessed 31 July 2024]. Reference Source 32212588

