# Supplementary material for: Severe, uncontrolled epilepsy in pregnancy: A population-based case-control study
Source: NIHR Open Res. 2025 Feb 12;4:53. Originally published 2024 Sep 24. [Version 2] doi: 10.3310/nihropenres.13743.2 (PMC11971622; doi:10.3310/nihropenres.13743.2)
Supplement: Peer review history

# Reviewer report: Reviewer response for version 2

**DOI:** 10.3310/nihropenres.14996.r34842

**Published:** 2025-04-04

**Authors:** Laura Kirkpatrick

**Affiliations:** 1 University of Pittsburgh, Pittsburgh, USA

Thank you for the opportunity to review your manuscript. This manuscript presents a case-control study of women with "severe, uncontrolled epilepsy" using a pragmatic case definition compared with an unmatched control group of women with epilepsy not meeting the case definition. The authors found that cases compared to controls were more likely to have been admitted for seizures the year before pregnancy, to report most recent seizure within 3 months of pregnancy, and to deliver before 37 weeks of pregnancy.

 The work is clearly and sufficiently reported, the statistical analysis is appropriate, and the conclusions are reasonable.

 My concerns pertain to the premise of the study and the pragmatic definition of "severe, uncontrolled epilepsy." Given the absence of a standard definition in the field for "severe, uncontrolled epilepsy," I am confused about why the investigators even pursued this line of inquiry, as opposed to selecting a more definitive and clear variable for the study. Their pragmatic definition was also difficult to implement, given that so many referred cases did not actually meet the case definition.

 I am generally concerned about the validity of this variable, and this is unfortunately not a fixable problem with this research.

Is the work clearly and accurately presented and does it cite the current literature?

Yes

If applicable, is the statistical analysis and its interpretation appropriate?

Yes

Are all the source data underlying the results available to ensure full reproducibility?

Yes

Is the study design appropriate and is the work technically sound?

No

Are the conclusions drawn adequately supported by the results?

Yes

Are sufficient details of methods and analysis provided to allow replication by others?

Yes

Reviewer Expertise:

epilepsy and reproductive health

I confirm that I have read this submission and believe that I have an appropriate level of expertise to state that I do not consider it to be of an acceptable scientific standard, for reasons outlined above.

# Reviewer report: Reviewer response for version 2

**DOI:** 10.3310/nihropenres.14996.r34893

**Published:** 2025-04-04

**Authors:** Masaraf Hussain

**Affiliations:** 1 All India Institute of Medical Sciences, Guwahati, Assam, India

The manuscript has elaborated the study with the results and analysis.

 1. Are there any confounders and biases in the study?

 2. Is the methodology reproducible?

 3. Does severe uncontrolled epilepsy have any relation with DRE (Drug resistant epilepsy?

 4. Will unmatched controls have any effect on the power of the study?

Is the work clearly and accurately presented and does it cite the current literature?

Yes

If applicable, is the statistical analysis and its interpretation appropriate?

I cannot comment. A qualified statistician is required.

Are all the source data underlying the results available to ensure full reproducibility?

Yes

Is the study design appropriate and is the work technically sound?

Yes

Are the conclusions drawn adequately supported by the results?

Yes

Are sufficient details of methods and analysis provided to allow replication by others?

Yes

Reviewer Expertise:

Dementia, Epilepsy

I confirm that I have read this submission and believe that I have an appropriate level of expertise to confirm that it is of an acceptable scientific standard, however I have significant reservations, as outlined above.

# Reviewer report: Reviewer response for version 2

**DOI:** 10.3310/nihropenres.14996.r34844

**Published:** 2025-03-20

**Authors:** Joan E Devin

**Affiliations:** 1 Royal College of Surgeons in Ireland, Dublin, Leinster, Ireland

Thank you for the opportunity to review this important paper comparing the characteristics, clinical management, and pregnancy outcomes in women with epilepsy in pregnancy with severe and controlled disease. Findings emphasise the need for targeted preconception care in women admitted for seizure management in the year prior to pregnancy, due to their increased risk of severe epilepsy in pregnancy and preterm birth. I note the previous reviewer’s comments, and amendments made by the authors in response.

 My additional comments are below:

  

- With no established definition for ‘severe and uncontrolled epilepsy in pregnancy, a pragmatic case definition was developed by the authors, which included treatment with three or more antiepileptic drugs simultaneously in pregnancy'. Please clarify if this definition included women who were prescribed 3 or more AEDs simultaneously at any one point in pregnancy, or if it reflects continous use. 
- Table 2 shows that 100% of the cases were known to epilepsy services at booking. As two of the cases were diagnosed with epilepsy during pregnancy, were they previously known to neurology/epilepsy services for other reasons?
- The numbers for the type of analgesia for labour and delivery in Table 4 appear to include the eight pregnancies that ended in miscarriage or termination (94 cases, 186 controls), which were not included in this analysis.
- Could the authors comment on the use of high-dose folic acid in the dataset if known, and in the cases and controls who had anomalies confirmed at delivery.
- The authors state that 29.0% of controls were not prescribed AEDs in pregnancy. Please also provide the proportions for AED monotherapy and polytherapy in this group.
- The Topiramate Pregnancy Prevention Plan for women with epilepsy has also been introduced since this study was conducted. Were any of the women in this study taking topiramate?

Is the work clearly and accurately presented and does it cite the current literature?

Yes

If applicable, is the statistical analysis and its interpretation appropriate?

Yes

Are all the source data underlying the results available to ensure full reproducibility?

Partly

Is the study design appropriate and is the work technically sound?

Yes

Are the conclusions drawn adequately supported by the results?

Yes

Are sufficient details of methods and analysis provided to allow replication by others?

Yes

Reviewer Expertise:

Drug utilisation in pregnancy; midwifery

I confirm that I have read this submission and believe that I have an appropriate level of expertise to confirm that it is of an acceptable scientific standard, however I have significant reservations, as outlined above.

# Reviewer report: Reviewer response for version 2

**DOI:** 10.3310/nihropenres.14996.r34727

**Published:** 2025-02-24

**Authors:** Neda Razaz

**Affiliations:** 1 Karolinska Institutet, Stockholm, Sweden

Thank you for addressing my comments, I still believe the statement of *Statistical significance *can be reduced in the manuscript and more emphasis should be on the direction of the association (e.g., no association instead of no statistically significant differences). Otherwise I have no further comment

Is the work clearly and accurately presented and does it cite the current literature?

Yes

If applicable, is the statistical analysis and its interpretation appropriate?

Partly

Are all the source data underlying the results available to ensure full reproducibility?

Partly

Is the study design appropriate and is the work technically sound?

Yes

Are the conclusions drawn adequately supported by the results?

Partly

Are sufficient details of methods and analysis provided to allow replication by others?

Yes

Reviewer Expertise:

Perinatal epidemiology

I confirm that I have read this submission and believe that I have an appropriate level of expertise to confirm that it is of an acceptable scientific standard.

# Reviewer report: Reviewer response for version 1

**DOI:** 10.3310/nihropenres.14926.r32980

**Published:** 2024-10-08

**Authors:** Neda Razaz

**Affiliations:** 1 Karolinska Institutet, Stockholm, Sweden

I read the manuscript with great interest. It is an important study with a unique and rich dataset, contributing valuable insights into the frequency of seizures among women with epilepsy—an area that is difficult to capture in studies using health administrative databases. Please find some comments below:

  

- Given that this is primarily a descriptive study with small sample sizes for most outcomes, I recommend removing **any** references to statistical significance from the Results and Discussion sections. For example, Table 6 shows differences between cases and controls in maternal morbidity, but there are only five cases in the control group, making adjusted analysis with more variables than outcomes implausible. Furthermore, in the Results section, it is indicated that there are no statistical differences in the odds for mode of delivery, whereas there is a clear difference in the rate of emergency CS among controls that is not being discussed.
- The stepwise regression might lead to overfitting and can also result in variables being included or excluded based on sample-specific data, which in this study has low power for some important variables. Additionally, the ordering of variables based on their temporal relationship could bias the association, with those closer to the pregnancy being more likely to affect the outcome, which may dilute other important associations.
- Please clarify what is meant by "other significant pregnancy outcomes" adjusted for in the models.
- What is the rationale for including individuals diagnosed with epilepsy after conception (two in cases, two in controls)?
- In Table 2, controls have a 60% rate of seizures in the year before pregnancy, indicating they are also a high-risk group. Additionally, the control group had more abnormalities than the cases, which could be discussed further.
- The number of mental health disorders is quite low in this cohort, which is inconsistent with previous findings in this population. Are these cases self-reported, and how is mental health captured in this cohort?
- References 27 and 26 don't seem relevant to the statement about pre-eclampsia and gestational diabetes rates being similar to the general population.

Is the work clearly and accurately presented and does it cite the current literature?

Yes

If applicable, is the statistical analysis and its interpretation appropriate?

Partly

Are all the source data underlying the results available to ensure full reproducibility?

Partly

Is the study design appropriate and is the work technically sound?

Yes

Are the conclusions drawn adequately supported by the results?

Partly

Are sufficient details of methods and analysis provided to allow replication by others?

Yes

Reviewer Expertise:

Perinatal epidemiology

I confirm that I have read this submission and believe that I have an appropriate level of expertise to confirm that it is of an acceptable scientific standard, however I have significant reservations, as outlined above.

# Response

**Published:** 2025-02-11

**Authors:** Ruth Tunn

**Affiliations:** NPEU, University of Oxford, Oxford, England, UK

*Thank you for your constructive comments. The authors have written the responses below, which I am submitting on their behalf. Ruth Tunn, Research Facilitator, National Perinatal Epidemiology Unit, University of Oxford.*

**Reviewer 1**

*I read the manuscript with great interest. It is an important study with a unique and rich dataset, contributing valuable insights into the frequency of seizures among women with epilepsy—an area that is difficult to capture in studies using health administrative databases. Please find some comments below:*

- * Given that this is primarily a descriptive study with small sample sizes for most outcomes, I recommend removing any references to statistical significance from the Results and Discussion sections. For example, Table 6 shows differences between cases and controls in maternal morbidity, but there are only five cases in the control group, making adjusted analysis with more variables than outcomes implausible. Furthermore, in the Results section, it is indicated that there are no statistical differences in the odds for mode of delivery, whereas there is a clear difference in the rate of emergency CS among controls that is not being discussed.*

**Response: **Thank you for your comments. We agree that the small number of events for some outcomes is a limitation of the study; as mentioned in the ‘strengths and limitations’ section of the Discussion, this is a consequence of low disease incidence and thus limitations on feasible sample size. This underscores the difficulty of investigating uncommon conditions in pregnancy and the value of the UKOSS platform in allowing a nationwide and prospective approach to data collection. Because of the small numbers involved, our estimates of association are relatively imprecise, evidenced by wide confidence intervals. However, the typical limitation of a small sample size would be insufficient power to detect genuine associations; the fact that we detected these patterns in a small sample suggests a genuine association.

 The odds of emergency caesarean section estimated in this study (9% [8/89] in cases versus 13.7% [25/183] in controls [95% CI 0.29-1.62]) are consistent with anything from a 71% reduction to a 62% increase in odds among cases. We therefore think that any discussion of the differences between the groups based on these data could be seen as speculative at best.

- *The stepwise regression might lead to overfitting and can also result in variables being included or excluded based on sample-specific data, which in this study has low power for some important variables. Additionally, the ordering of variables based on their temporal relationship could bias the association, with those closer to the pregnancy being more likely to affect the outcome, which may dilute other important associations.*

**Response: **We consider that the adjusted associations for preterm birth and maternal morbidity have not been diluted, as evidenced by the increased magnitude of the adjusted association versus the unadjusted. This increased magnitude after adjustment for potential confounders could be explained by the small number of outcomes among controls for preterm birth, and among both cases and controls for maternal morbidity. However, it must be noted that preterm birth remained significant after adjustment for covariates, and maternal morbidity was non-significant in both the adjusted and unadjusted models. The magnitude of the association observed for newborn morbidity reduced after adjustment for covariates not on the causal pathway, especially gestational age at birth and confirmed congenital abnormality, as one would expect in an adjusted analysis.

- *Please clarify what is meant by "other significant pregnancy outcomes" adjusted for in the models.*

**Response: **We apologies for the lack of clarity. ‘Significant pregnancy problems’ relates to question 4.13 on the data collection form, ‘Were there other problems in this pregnancy?’. Potential conditions are listed in the Definitions section at the end of the data collection form, list number 2.

 The Methods includes a link to the data collection form, and we have updated the relevant text in the ‘statistical methods’ section and the footnote to Table 6 to clarify.

- *What is the rationale for including individuals diagnosed with epilepsy after conception (two in cases, two in controls)?*

**​​​​​​​Response: **Our study aimed to explore severe epilepsy during pregnancy. There is potential for valuable lessons to be learned from the experiences of woman who were not diagnosed until after they became pregnant, so we did not consider it appropriate to exclude them.

- *In Table 2, controls have a 60% rate of seizures in the year before pregnancy, indicating they are also a high-risk group. Additionally, the control group had more abnormalities than the cases, which could be discussed further.*

**​​​​​​​Response: **We apologies that ‘Predominant seizure type during the 1 year pre-pregnancy’ in Table 2 was an inaccurate description of the variable in question. This variable relates to question 3a.5 on the data collection form, ‘What type of seizures does the woman have?’ Thus, the 59.7% of control women with convulsive seizures refers to the predominant seizure type in these women throughout their life pre-pregnancy, rather than in the preceding year only. We have updated the variable in Tables 2 and 5, and the corresponding main text, to clarify.

 As indicated under ‘Most recent seizure before pregnancy’ in Table 2, 43/186 women in the control group (18 + 12 + 13), or 23.1%, experienced their most recent pre-pregnancy seizure within the 12 months preceding pregnancy. This suggests considerably lower risk compared with the figure of 66% for cases.

 The number of confirmed fetal abnormalities was slightly higher among controls (n = 5) compared with cases (n = 1), even after taking into account the larger denominator for controls. However, there was no obvious pattern in the types of abnormalities, and the very small numbers involved mean that any attempt to draw conclusions about differences between cases and controls would be entirely speculative.

- *The number of mental health disorders is quite low in this cohort, which is inconsistent with previous findings in this population. Are these cases self-reported, and how is mental health captured in this cohort?*

**​​​​​​​Response: **We apologies for any confusion. This variable refers to newly recognised mental health problems in pregnancy. We have clarified this in Table 3 and the main text. Nineteen of the women with uncontrolled epilepsy (20.2%) were known to have mental health problems prior to pregnancy. We have added this information to the “Characteristics of cases and controls” section of the results text.

- *References 27 and 26 don't seem relevant to the statement about pre-eclampsia and gestational diabetes rates being similar to the general population.*

**​​​​​​​Response: **The NICE guidance (reference 27) indicates that ‘approximately 700,000 women give birth in England and Wales each year, and up to 5% of these women have either pre-existing diabetes or gestational diabetes.’ In our study, gestational diabetes was reported in 5.3% of cases and 2.7% of controls which, in the context of the small sample size discussed previously, does not suggest any remarkable difference between groups or deviation from the general population.

 The NHS guidance for maternal blood pressure monitoring (reference 26) is grounded in the evidence that ‘hypertensive disorders occur in 8 to 10% of all pregnancies’. In this study, pre-eclampsia was reported in 2.1% of cases and 5.4% of controls which, again, we do not consider meaningfully different between groups. If anything, the incidence is slightly lower in our cohort than in the general population. However, the small sample may again be operating as a factor here.
